# Knowledge gaps and future perspectives for the definition, diagnostics and treatment of isolated locoregional recurrent breast cancer

**DOI:** 10.1016/j.breast.2026.104945

**Published:** 2026-10-21

**Authors:** Tess I.M. Snellen, Charlotte M. Vervenne, Vincent O. Dezentjé, Terry Wiersma, Astrid N. Scholten, Esther H. Lips, Marie-Jeanne T.F.D. Vrancken Peeters

**Affiliations:** a Department of Surgical Oncology, Netherlands Cancer Institute – Antoni van Leeuwenhoek, Plesmanlaan 121, 1066 CX, Amsterdam, the Netherlands; b Department of Medical Oncology, Netherlands Cancer Institute – Antoni van Leeuwenhoek, Plesmanlaan 121, 1066 CX, Amsterdam, the Netherlands; c Department of Radiation Oncology, Netherlands Cancer Institute – Antoni van Leeuwenhoek, Plesmanlaan 121, 1066 CX, Amsterdam, the Netherlands; d Department of Molecular Pathology, Netherlands Cancer Institute – Antoni van Leeuwenhoek, Plesmanlaan 121, 1066 CX, Amsterdam, the Netherlands; e Department of Surgery, Amsterdam University Medical Center, Meibergdreef 9, 1105 AZ, Amsterdam, the Netherlands

**Keywords:** Isolated locoregional recurrence, Breast cancer, Staging, Re-irradiation, Axillary management, Systemic therapy, Treatment de-escalation

## Abstract

Isolated locoregional recurrence (ILRR) of breast cancer (BC) is associated with a poor prognosis and a high risk of distant metastasis (DM) and death. As ILRR occurs in around 0.5-1.5% of BC patients annually, and the overall prevalence of primary BC is high, the absolute number of ILRR BC patients is substantial. Diagnosing a second BC event can be challenging, particularly distinguishing between a true recurrence (TR) and a second primary (SP). This distinction is clinically relevant, as TRs are associated with a poorer prognosis, especially when presenting as regional recurrences, and require a more challenging therapeutic approach compared to SPs.

The radiologic work-up of suspected BC recurrence depends on the tumor location (breast, chestwall, or regional), clinical presentation, prior treatment characteristics, and should include systemic staging given the substantial risk of synchronous distant metastases. Robust scientific evidence and guidelines on locoregional and systemic treatment of ILRR of BC are lacking. Consequently, ILRR management currently relies on expert opinion, and therefore may vary between institutions. This review aims to provide an overview of current knowledge on definition, diagnostic workup, staging, locoregional treatment, and systemic treatment of ILRR. Furthermore, ongoing clinical research and future research perspectives to improve the diagnostic work-up and multimodality treatment of patients with ILRR will be outlined.

## Highlights

• Definition and standardized diagnostic work up of ILRR are lacking.
• Multidisciplinary ILRR treatment is essential, but dedicated guidelines are lacking.
• Treatment de-escalation seems feasible in selected ILRR patients.
• Re-irradiation is effective in selected patients, but optimal techniques remain undefined.
• Evidence for systemic therapy in ILRR is limited; prospective trials are needed.

## Graphical abstract

**Figure:**
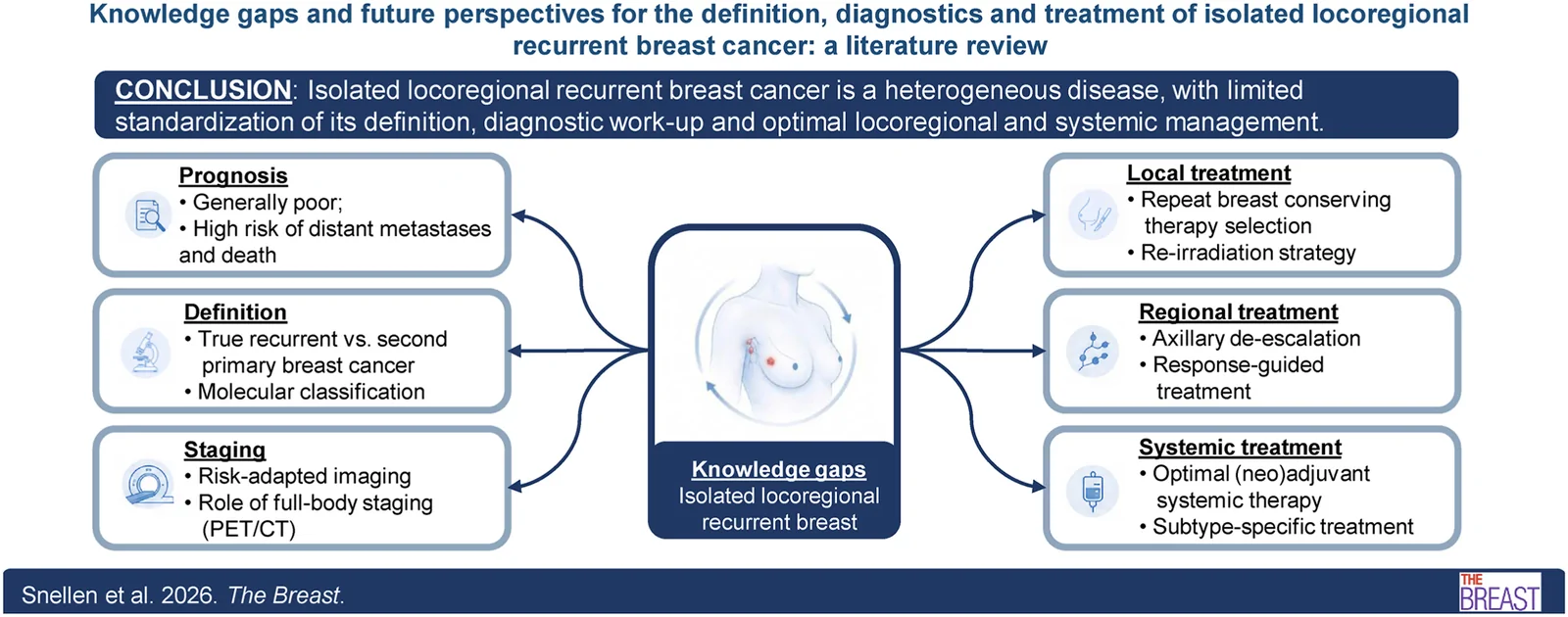

## 1 Introduction

An isolated locoregional recurrence (ILRR) of breast cancer (BC) is defined as reappearance of disease in the ipsilateral breast, chest wall, and/or in the regional lymph nodes (axillary, supraclavicular, infraclavicular or internal mammary chain (IMC)), without evidence of distant metastases (DM). The cumulative incidence of ILRR after primary BC treatment is 5-10% after 10 years of follow-up [1], [2], [3], [4]. With around 2.3 million new BC patients per year [5], the absolute number of patients diagnosed with an ILRR is substantial.

Prognosis after ILRR is generally poor, with increased risks of subsequent DM and death [2], [6], [7]. However, ILRRs vary in presentation and prognostic characteristics, ranging from small isolated in-breast recurrence to regional nodal relapse, which are two scenarios with substantially different prognoses. Geurts et al. reported subsequent DM in 22% of patients after an isolated local BC recurrence and 43% after regional recurrence [8]. These differences in disease presentation influence staging strategies and extent of locoregional and systemic therapeutic approach underscoring the complexity of ILRR management.

Diagnosing an ILRRis challenging, particularly when distinguishing true recurrence (TR) from second primary (SP) BC. This distinction is clinically relevant, as TRs are associated with a poorer prognosis [2], [6], [9], [10] and require more challenging treatment compared to SPs. Currently, this distinction primarily relies on unvalidated clinicopathological criteria, such as tumor location, histological subtype, grade, hormone receptor and human epidermal growth factor receptor 2 (HER2) status, disease free interval (DFI), and prior margin status [9], [10], [11], [12], [13]. Molecular clonality analysis (MCA) is occasionally performed, but consensus is lacking regarding its clinical indications and optimal MCA technique.

Accurate staging of ILRR is essential to guide locoregional treatment and exclude DM. However, scientific evidence and clinical guidelines on optimal staging and subsequent locoregional and systemic treatment are limited and research in this field has progressed slowly. Existing studies are small and inconsistent in ILRR definitions, limiting generalizability and preventing reliable comparison across treatment strategies. Consequently, ILRR management currently relies on expert opinion (multidisciplinary teams) and therefore varies between institutions.

This review summarizes current guideline recommendations (Table 1) and the available scientific evidence on ILRR BC, while identifying key knowledge gaps. We also provide an overview of ongoing clinical research and outline future research perspectives to improve the diagnostic work-up and multimodality treatment of patients with ILRR.

**Table 1:** Overview of international guideline recommendations for the diagnosis and treatment of isolated locoregional recurrent breast cancer.

Guideline	Version/Year	Staging	Local treatment	Regional treatment	Systemic treatment
NCCN [14]	**V4.2026**	**Standard** - CT thorax + CT abdomen with contrast OR - MRI with contrast - MRI-brain (if suspicion CNS symptoms) - MRI-spine if back pain - Bone scan OR - Sodium fluoride PET/CT **Additional** In certain (unspecified) circumstances: - FDG-PET/CT OR - FES-PET/CT for ER + disease and lobular histology.	**Prior BCS** - Repeat BCS OR - salvage mastectomy Without prior RT - RT after repeat BCS, and if indicated after mastectomy Prior RT - Repeat RT if feasible* and indicated *Consider in selected patients:* (who decline mastectomy and otherwise meet consensus criteria for RT omission or PBI): - Repeat BCS + adjuvant PBI (limited data) **Prior mastectomy** - Surgical resection (if feasible) Without prior RT - Consider PMRT Prior RT: - Repeat RT (if feasible* and indicated) *Consider prior RT to the area and the risk of late normal tissue toxicity after planned re-RT.	**cN0** - Consider surgical axillary staging: • Prior SLNB: repeat SLNB • Prior ALND: repeat SLNB + lymphscintigraphy **cN+** Prior SLNB - Surgical resection with ALND if feasible Prior ALND - Surgical resection of gross disease ± ALND if feasible Without prior RT: - RT Prior RT: - Re-RT, if feasible*. **Extra-axillary lymph nodes** Without prior RT: - RT (comprehensive or localized) After prior RT: - Re-RT, if feasible*.	- Consider neoadjuvant systemic therapy in all cases. - Consider appropriate adjuvant systemic therapy as clinically indicated.
ESMO [15]	**2021**	No specific recommendations for ILRR BC identified			
AGO [16] AGO grade of recommendation: ++: Strongly recommended +: Recommended +/−: No clear recommendation -: Not recommended --: Strongly not recommended	**2025**	**Standard** - CT thorax/abdomen/pelvis - Bone scan **Additional** In certain (unspecified) cases: - Whole body MRI OR - FDG-PET/CT	**Prior BCS** - Salvage mastectomy **++** OR - repeat BCS + PBI **+** (consider DFI, tumor size, risk factors for relapse) OR - repeat BCS **+/−** Without prior RT - WBRT **++** Prior RT - Re-RT (PBI) **+** **Prior mastectomy** - Complete excision **++** Without prior RT - RT chestwall, consider regional RT **+** Prior RT - re-RT (R0 **+/−**, R1 **+**, hyperthermia regional **+/−**)	**cN0** • Prior SLNB: repeat SLNB **-** • Prior ALND: surgical axillary intervention **-** **cN+** - Excision of suspicious lymph nodes **+** OR - Axillary lymph node dissection **+/−** Without prior RT: - RT of axilla after R0 **+** After prior RT: - RT of axilla **+/−**	**ER-positive** - ET **++** **ER-negative** (first relapse) - ChT **+** (consider neoadjuvant) **HER2-positive** - ChT and HER2-targeted **+** **Consider:** - ER, PR, HER2, DFI, pre-treatment-comorbidities and patient's preference.
NABON [17]	**2017**	**Standard** Mammography US MRI when the above are inconclusive **Additional** In case of M1 suspicion or abnormalities on conventional imaging: FDG-PET/CT Consider: - Contralateral mammography	**Prior BCT** - Salvage mastectomy OR - repeat BCS + re-RT (in selected patients) **Prior mastectomy** - Surgical resection Without prior RT - High dose RT (50Gy 5 weeks) Prior RT - Low dose re-RT + hyperthermia	**cN+** - Surgical resection Without prior RT - High dose RT (50Gy 5 weeks) After prior RT - Low dose re-RT + hyperthermia **Supracalvicular lymph nodes** - Radiotherapy (most effective)	**ER-positive** In case of aggressive tumor characteristics or no ChT for prior disease: - Adj ChT before start ET **ER-negative** - Adj ChT .
NICE [18]	**2025**	No specific recommendations for ILRR BC identified			
ASCO [19], [20], [21]	**Multiple**	No specific recommendations for ILRR BC identified			

Abbreviations: AGO, Arbeitsgemeinschaft Gynäkologische Onkologie; ALND, axillary lymph node dissection; ASCO, American Society of Clinical Oncology; BCS, breast-conserving surgery; ChT, chemotherapy; cN+, clinical node-positive; cN0, clinical node-negative; CNS, central nervous system; CT, computed tomography; DFI, disease-free interval; ER, estrogen receptor; ESMO, European Society for Medical Oncology; ET, endocrine therapy; FDG, fluorodeoxyglucose; FES, 16α-18Ffluoro-17β-oestradiol; Gy, Grey; HER2, human epidermal growth factor receptor 2; ILRR, isolated locoregional recurrence; MRI, magnetic resonance imaging; NABON, Netherlands Federation of Cancer Patient Organisations; NCCN, National Comprehensive Cancer Network; NICE, National Institute for Health and Care Excellence; PBI, partial breast irradiation; PET/CT, positron emission tomography/computed tomography; PMRT, post-mastectomy radiotherapy; PR, progesterone receptor; R0, free surgical margins; R1, microscopic positive margins; RT, radiotherapy; SLNB, sentinel lymph node biopsy; US, ultrasound; WBRT, whole breast radiotherapy.

## 2 Methods

This narrative review was based on a literature search performed in the MEDLINE database covering the period from January 2000 to September 2024. The search strategy included terms related to “locoregional recurrence” and “breast cancer”, combined with “diagnostic”, “prognostic”, “surgical”, “radiotherapy”, and “systemic treatment”. Eligible studies included original articles and reviews. Titles and abstracts were screened using Rayyan software to identify studies relevant to the main topics of this review: definition, staging, locoregional treatment, and systemic treatment of ILRR. Additional relevant publications were identified through cross-referencing and related-article searches. Published international guidelines and consensus recommendations were also reviewed to summarize current recommendations for the diagnosis and management of ILRR. The final selection of references was based on their relevance to the topics addressed in this narrative review.

## 3 Definition of ILRR

ILRR BC either presents a TR, due to outgrowth of residual primary tumors cells, or a SP. This distinction is clinically relevant as TRs are associated with a higher risk of DM and death [2], [6], [9], [10]. A TR may also have developed resistance to prior systemic treatment, limiting subsequent treatment options. Despite its clinical relevance [10], there is no consensus how to distinguish TR from SP. Current practice mostly relies on expert opinion using clinicopathologic variables.

### 3.1 Clinicopathologic variables

Various unvalidated classification methods are available using clinicopathologic criteria such as tumor location, histological subtype, tumor grade, hormone receptor and HER2 status, and prior margin status [9], [11], [12], [13], [22]. Jobsen et al. demonstrated that survival differences between TR and SP varied depending on the classification method used [10], underscoring their limited reliability and lack of interchangeability. Furthermore, some of these characteristics may have limited general applicability. Recurrent tumor location confined to the primary tumor bed (same quadrant, <3 cm radius [10]) is insufficient as ductal tumor cells may migrate [23], primary tumors can be multifocal or multicentric [23], and tissue displacement after prior breast conserving surgery (BCS) may influence local ILRR location. Additionally, these clinicopathologic classification methods assume concordant receptor status between primary and recurrent tumors. However, receptor discordance may reflect subclone outgrowth from a heterogeneous primary tumor or clonal tumor evolution [24], [25], [26], [27]. Furthermore, DFI is not incorporated in these methods, despite evidence suggesting that a short DFI, especially in HER2-positive and triple-negative ILRR, can be an indicator of TR [9], [28], [29].

Thus, while combining clinicopathologic variables may provide the most accurate prediction of TR, their relative individual contribution likely varies across molecular subtypes. Furthermore, their predictive performance appears limited compared with MCA [28], [29], [30], [31], [32].

### 3.2 Molecular testing

Molecular clonality analysis assesses clonal relatedness between primary tumors and ILRR by identifying shared somatic alterations. While previously loss of heterozygosity (LOH) analysis was used for clonal assessment [33], nowadays most labs rely on next-generation sequencing (NGS) based technologies, such a mutation panel sequencing [34], copy number variation sequencing (CNVseq) [35] or whole exome sequencing (WES) [36].

While WES is most informative, its high costs [37] and technical demands currently limit routine clinical use. Although all approaches have potential to establish clonal relatedness, the optimal method for clinical implementation remains undefined. Prior studies were limited by small sample sizes and technical issues, such as poor tissue quality [22], [28], [29], [30], [31], [32], [38], [39], [40], [41]. In addition, panel sequencing may be inconclusive due to low tumor mutational burden and the limited amount of hotspot mutations detected in BC [42]. Furthermore, common hotspot mutations (e.g., in PIK3CA [43]) may also occur independently in unrelated tumors, limiting definitive assessment of clonality [44].

Preliminary data on MCA use from a recent real-world cohort from our institute (n = 85) were presented at a scientific congress. Until 2017, LOH analysis was most commonly used, hereafter, mutation panel sequencing was most frequently used (54%), whereas CNV analysis, used in 15% of patients, achieved the highest success rate with the most conclusive result (93%) [45]. In this study we compared MCA-based classification with the Jobsen Morphology method [10] and observed a substantial discordance (30%) in defining a second event as TR or as SP.

Beyond qualitative assessment of shared genomic alterations, MCA can be applied in statistical models to quantify the likelihood of a clonal relationship. Although several statistical models have been developed [42], [44], [46], they are not yet used in clinical practice.

Overall, MCA is considered more informative than clinicopathologic classification methods for assessing relatedness between the primary and secondary tumor pairs [11], [38], [45]. Its current clinical value lies in guiding treatment decisions and prognostic assessment in patients with ipsilateral second BC. While molecular testing is increasingly integrated into stage IV BC to inform targeted therapies, its application in ILRR remains limited. Broader implementation of MCA in second BC events should be considered, with technique selection guided by cost and feasibility.

### 3.3 Future perspectives

Future research should determine the optimal MCA technique and establish clear clinical indications for clonality testing. MCA provides clinically relevant differentiation between TR and SP when treatment decisions depend on this distinction. Accordingly, as the accuracy and accessibility of clonality testing continue to improve, we advocate for clear thresholds and standardized molecular approaches to enable routine use in clinically indicated cases.

Comparative research should determine which MCA technique provides the best balance between accuracy, generalizability and cost-effectiveness in clinical practice. Combining MCA techniques, such as mutation panel sequencing and CNVseq analysis, may also enhance diagnostic accuracy. Furthermore, clinicopathologic criteria should be validated in large, unselected cohorts of patients with ipsilateral second BCs against a molecular gold standard, ideally genome-wide MCA with standardized interpretation or within a statistical model. These validated criteria could be particularly useful in settings with limited access to MCA.

Looking ahead, artificial intelligence may support the assessment of clonality by integrating histopathological and molecular data, potentially improving diagnostic accuracy while reducing the need for extensive genomic testing. Ultimately, a broadly applicable classification framework integrating molecular, morphological and clinical data is needed to consistently define TRs, ensuring consistency in research, refine prognostic assessments, and guide treatment. Continued validation of such multimodal approaches will contribute to more personalized care for patients with recurrent BC.

## 4 Staging

When ILRR is suspected, the radiologic work-up depends on recurrent tumor location (breast, chest wall, regional lymph nodes) and clinical presentation.

Current staging guidelines do not distinguish ILRR from de novo metastatic disease, and therefore often recommend a full-body radiological assessment, including bone scans, chest and abdominal computer tomography (CT)-scans and brain magnetic resonance imaging (MRI) [14], [16]. However, ILRR represents a fundamentally different clinical entity with curative intent. And as ILRR varies in its clinical presentation, applying extensive staging to all ILRR cases may result in unnecessary diagnostic burden without clear clinical benefit.

### 4.1 Locoregional staging by imaging

After prior breast conserving treatment (BCT), radiologic work-up should include mammography and breast and axillary ultrasound (US) with histopathologic confirmation of suspected lesions. Furthermore, the addition of MRI improves the accuracy of local tumor size estimation and detection of multifocality [47], [48], which is particularly relevant in patients eligible for repeat BCS. In post-mastectomy patients, MRI provides substantial value, besides physical examination and US, to assess the recurrent lesions size and spread [49], especially in small (<1 cm) and asymptomatic lesions [50], demonstrating superior sensitivity and specificity (both 100% [49], [51]).

Regional recurrences should initially be assessed with axillary and periclavicular US [51]. In the primary setting, MRI demonstrates diagnostic accuracy comparable to US for axillary staging (sensitivity 79%, specificity 82% [52]). Although dedicated data in the recurrent setting are lacking, the American College of Radiology (ACR) guidelines consider MRI appropriate for preoperative axillary staging [53].

(18) F-fluorodeoxyglucose positron-emission tomography/computed tomography (FDG PET/CT) shows high accuracy for detecting regional nodal recurrences, with pooled sensitivity and specificity exceeding 90% [17], and is generally recommended as an adjunct to conventional locoregional imaging (mammography, US, MRI) [17], [54], [55].

In the primary setting, FDG PET/CT detects additional regional lymph nodes in 12% of patients following conventional locoregional staging [56]. The impact of FDG PET/CT on regional staging in the recurrent BC setting remains unknown. Haarsma et al. did demonstrated that, in patients with clinically node negative (cN0) ILRR on FDG PET/CT, repeat sentinel lymph node biopsy (SLNB) can safely be omitted [57], suggesting that elaborate imaging may reduce the application of invasive treatment. Additionally, FDG PET/CT may also identify aberrant lymphatic drainage following prior axillary surgery, which can result in disease spread beyond conventional locoregional imaging areas [55]. Importantly, contralateral regional recurrences after prior axillary surgery can still be managed as ILRRs and treated with curative intent rather than metastatic disease [58], [59]. It should be noted that FDG PET/CT may be less informative in certain tumor subtypes, such as lobular cancer, due to their relatively low FDG avidity [60].

### 4.2 Metastatic work-up

As ILRR is associated with a high risk of distant disease, the question remains whether all ILRR patients should undergo full body staging [61]. Current guidelines often recommend extensive imaging, including FDG PET/CT, CT and/or bone scans (Table 1), with histopathological confirmation of suspicious lesions whenever feasible. However, ILRR is a heterogeneous disease, ranging from small local to isolated regional recurrences. Consequently, not all patients may benefit from elaborate metastatic work up. Especially in patients with low-risk features (e.g. small tumor size, cN0 disease, estrogen receptor (ER)-positivity, and low grade).

Clinical guidelines recommend FDG PET/CT in ‘certain circumstances’, but do not further specify what these circumstances are [14], [16]. The Dutch national guideline advises FDG PET/CT in cases of suspected metastatic disease or other abnormalities on conventional imaging [17], and states FDG PET/CT has largely replaced these modalities in clinical practice. The EANM-SNMMI guideline recommends its use in patients with signs or symptoms suggestive of metastatic disease and substitute CT and/or bone scans for the detection of bone lesions [55]. FDG PET/CT enables earlier detection of bone metastases and identification of lesions non-measurable on CT [62], with lesion-based sensitivity comparable to combined bone scan and low-dose CT (98% vs. 99%, respectively), and superior sensitivity to either modality alone (low-dose CT (80%); bone scan (76%)) [63].

Several studies report a substantial impact of FDG PET/CT on treatment strategies, around 51-69% of ILRR patients, by up- or downstaging of the recurrent disease [60], [64], [65], [66], [67], [68], [69]. In a small cohort (n = 56), FDG PET/CT detected additional metastatic lesions in ∼45% of cases following conventional imaging and altered disease management (e.g. curative to palliative) in around half of patients [70]. However, all studies included all types of ILRR patients (e.g. isolated local and regional disease), and therefore ILRR patient selection for FDG PET/CT remains unclear [60].

In ER-positive ILRR (and DM), 16α-18Ffluoro-17β-oestradiol (FES) PET/CT enables assessment of ER expression. In patients with ER-positive ILRR and low FDG avidity, FES PET/CT may improve diagnostic accuracy and inform treatment decisions, supporting a more personalized management of ILRR [71], [72]. According to the National Comprehensive Cancer Network (NCCN) guideline, FES PET/CT for ER-positive (especially lobular) histology should be considered [14].

### 4.3 Future perspectives

Future research should develop risk prediction models based on patient and disease characteristics to identify patients at high risk of synchronous DM during ILRR diagnosis. Such models may facilitate evidence-based patient selection for comprehensive diagnostic work-up, tailored to ILRR risk profiles. For example, low-risk patients may not require additional staging, whereas intermediate-risk patients could undergo FDG PET/CT when available, or alternatively chest and abdominal CT, and high-risk patients may benefit from FDG PET/CT as a first-line modality.

The value of FDG PET/CT in nodal assessment remains unclear. Although retrospective evidence suggests that repeat SLNB may be safely omitted in FDG PET/CT-defined cN0 patients, implementation in clinical practice is inconsistent. For distant staging, FDG PET/CT may outperform conventional imaging, yet the patient subgroups with the greatest clinical benefit remain undefined. It is also unclear whether FDG PET/CT should be reserved for cases with suspicious findings on conventional imaging or as a first-line modality. Conversely, a subset of ILRR may be non-FDG-avid (e.g. lobular cancer), warranting standard locoregional imaging or further advances in nuclear medicine. FES PET/CT for ER-positive ILRR, HER2-directed imaging with 89Zr-Trastuzumab PET/CT for HER2-positive disease, and fibroblast activation protein inhibitor (FAPI)-PET offer biologically specific imaging that may refine characterization of ILRR.

## 5 Locoregional treatment: surgery and radiation therapy

### 5.1 Local treatment of recurrent disease

#### 5.1.1 After prior breast conservative treatment

Currently, salvage mastectomy remains the recommended local treatment for ILRR after prior BCT (BCS and radiotherapy) [14], [16], [17]. However, repeat BCT is a feasible alternative in selected patients, with comparable oncologic outcomes [1], [73], [74], [75], [76], [77], [78], [79], [80], [81], [82], [83], [84], [85]. In a large retrospective GEC-ESTRO study (n = 1327), all women diagnosed between January 1995 and June 2017 with an ILRR occurring after BCT of the primary were included [75]. Eligible patients had T1-2 tumor, a DFI of at least 12 months and no evidence of skin involvement, DM or history of contralateral BC. Patient selection for conservative treatment considered tumor stage, multicentricity, breast size, potential long-term effects of the first conservative treatment (according to the GEC-ESTRO accelerated partial breast irradiation (APBI) classification [86]). Negative resection margins were mandatory. No differences in oncologic outcomes between salvage mastectomy and repeat BCT with brachytherapy were observed, with comparable second recurrence rates and 5-year overall survival (OS) (2.3% vs. 2.8%, p = 0.40; 88% vs. 87%, respectively) [75]. Physician-reported cosmetic outcomes after repeat BCT (n = 377) were favorable in 212 patients (56%) (Harvard criteria [87]), with 80% rated as good or excellent.

Neoadjuvant systemic therapy (NST) has enabled tumor downstaging and increased conversion rates from mastectomy to BCT in primary BC [88], [89], [90], [91]. Although NST may similarly facilitate repeat BCT in ILRR, the optimal NST regimen per subtype, the frequency of local re-staging, and favorable patient and tumor characteristics associated with successful ILRR (local) down staging remain undefined.

No standardized guidelines exist to guide patient selection for repeat BCT. A systematic review by Walstra et al. (n = 34 studies) identified key considerations including patient preference, acceptable late-toxicity from prior radiotherapy allowing safe re-irradiation, and a favorable tumor-to-breast ratio ensuring acceptable cosmetic outcomes [78]. Additional factors include DFI, tumor size, and recurrent tumor location [74], [77], [79], [92], [93], [94]. Indications for salvage mastectomy include unfavorable histological factors, significant late toxicity from prior radiotherapy, breast size or patient preference [86].

Recently, an international Delphi consensus study further defined patient selection for repeat BCT, identifying a DFI ≥ 60 months, favorable tumor-to-breast ratio, clear margins, absence of severe late toxicities (grade ≥3), and low TAM score (a prognostic score for repeat BCT with partial breast irradiation (PBI) [76]) as key selection criteria [95]. Notably, premenopausal status and HER2-positive and triple-negative subtypes were not considered absolute contraindicated by the panelists.

These findings highlight the importance of individualized treatment selection. Shared decision-making remains essential, as patient perspectives and expectations are important determinants in the choice between repeat BCT and salvage mastectomy [95], [96]. In clinical practice, histopathological factors should guide primary decision-making, complemented by consideration of breast size, late radiotherapy toxicity, and patient preference.

#### 5.1.2 Re-irradiation after prior breast conservative treatment

Re-irradiation is a key component in selecting patients for repeat BCT. A national survey among Dutch breast surgeons and radiation oncologists identified concerns regarding re-irradiation toxicity as the main concern to apply repeat BCT [97]. Re-irradiation can be delivered using multicatheter interstitial brachytherapy (MIBT), intraoperative radiation therapy (IORT), and external beam radiotherapy (EBRT) [78]. Although MIBT is the most extensively studied and historically applied modality [83], [94], [98], EBRT is more widely available and routinely implementable in clinical practice. Reported rates of ≥3 grade radiotherapy toxicity range from 0 to 12.1% across techniques [75], [76], [99], [100], [101], [102], [103], [104]. The recent Delphi consensus similarly favored MIBT, while considering EBRT a reasonable alternative [95]. Despite toxicity concerns, re-irradiation after BCS is essential to improve local control [73], [78] and increase OS [92], consistent with the primary setting. Advances in primary radiotherapy, including dose de-escalation, hypofractionation and improved technical advances for normal tissue sparing, have reduced radiotherapy toxicity and thereby created more opportunities for safe re-irradiation. However, the optimal re-irradiation dose and fractionation remain undefined and are currently extrapolated from primary treatment protocols or hyperthermia-based regimens. Accordingly, the Delphi panel supported extrapolation of dose constraints and contouring principles from the primary setting to guide re-irradiation planning, while favouring partial breast techniques in this setting [95].

PBI limits the irradiated breast tissue volume and thereby reduces the risk of late radiation toxicity [105], [106], while also limiting radiation exposure to organs at risk [107], [108], [109], making it a feasible re-irradiation strategy for local ILRR. In a prospective single-arm trial (n = 65) on repeat BCT with postoperative PBI for local ILRR, Arthur et al. reported a 5-year BC recurrence rate of 5%, and grade 3 toxicity in 7% of patients [100]. Furthermore, as PBI is increasingly used in the primary setting, this may preserve normal tissue and facilitate subsequent re-irradiation in the recurrent setting.

Risk stratification tools may support patient selection. Eligibility criteria and risk factors for PBI in primary BC from multiple guidelines are summarized in Supplementary Table 1 [110], [111], [112], [113], [114], while Table 2 outlines criteria for recurrent BC from literature. Although the GEC-ESTRO and ASTRO PBI risk classification have recently been updated with broadened eligibility criteria (Supplementary Table 1), earlier criteria developed for the primary setting have demonstrated utility in ILRR. Montange et al. reported a 6-year third ipsilateral breast tumor-free survival rate of 100% in the low-risk and 95.8% in the intermediate-risk group classified according to the original GEC-ESTRO [110] and ASTRO [111] PBI classifications [86]. In addition, the TAM prognostic scoring system [76] estimates the risk of a second local recurrence after repeat BCT with APBI, incorporating time interval (T) from initial to repeat surgery, APBI (A) eligibility criteria based on the original GEC-ESTRO patient selection guideline [110], and molecular (M) classification (ER and HER2 status [115]). Patients are stratified into low-, intermediate-, or high-risk group. The TAM score was an independent prognostic factor for nearly all oncologic outcomes (p < 0.001), highlighting its potential value for risk stratification.

**Table 2:** Eligibility criteria and risk factors for partial breast irradiation in isolated locoregional recurrent breast cancer.

	Montagne et al., 2019 [86]	Hannoun-Levi et al., 2023 [76]
**Eligibility criteria**	**Consider** • Prior total RT dose • Remaining breast volume • Cosmetic impact • Patient's preference	**Repeat BCT contraindications** • Multicentric tumors • Major breast deformations • Patient preference
**Risk factors**	• LVI • Extensive intraductal component • High Ki67 • Poor histological differentiation (e.g. high tumor grade)	• LVI • extensive intraductal component • High Ki67 • Grade 3 • HER2 overexpression • TNBC molecular status

Abbreviations: BCT, breast conserving therapy; HER2, human epidermal growth factor receptor 2; LVI, lymphovascular invasion; RT, radiotherapy; TNBC, triple negative breast cancer.

To further optimize treatment, PBI could be administered pre-operatively, allowing for more accurate tumor delineation, enabling smaller treatment volumes and removal of irradiated tissue, thereby reducing radiation toxicity and improving cosmetic outcomes. Although currently limited to the primary BC setting, preoperative PBI demonstrated low rates of severe toxicity, favorable cosmetic outcomes, and promising oncological 5-year oncological outcomes [116], [117], [118], [119].

Proton radiotherapy is another potential re-irradiation strategy that reduces the dose to organs at risk due to the absence of an exit dose and offers superior dose-conformity compared to photon radiotherapy, while maintaining a curative dose to the tumor bed [120], [121], [122], [123], [124]. In a cohort study (n = 46), proton re-irradiation had a 2-year distant disease-free survival (DDFS) and OS were 92.0% and 93.6%, respectively [120], with minimal high-grade toxicity. However, larger studies with longer follow-up are needed [121].

#### 5.1.3 After prior mastectomy

In patients with ILRR after prior mastectomy, local surgical resection is recommended when feasible [14]. For extensive disease, radical chest wall resection may be required [125], [126]. For unresectable disease, NST should be considered with the potential for subsequent resection based on treatment response, or definitive radiotherapy alone [85], [127]. Multimodality treatment combining local surgery and (re-)irradiation improves local disease control and survival [127], [128], [129]. In previously irradiated patients, the decision to re-irradiate should balance recurrence risk against potential toxicity, considering prior radiation dose and pre-existing treatment-related toxicity [14], [79], [94], [127], [129], [130]. Reported rates of ≥ grade 3 toxicity after chest wall re-irradiation are acceptable, ranging from 8.9 to 17% [131], [132].

To reduce toxicity, re-irradiation is frequently combined with hyperthermia, enabling dose reduction while maintaining treatment efficacy [127]. In a systemic review, Datta et al. reported acute grade 3-4 toxicities in 14.4% of patients and late toxicities of 5.2% with this approach [133]. In addition, chemoradiotherapy has also been explored for unresectable disease, showing durable local control in small studies, particularly in radiotherapy-negative patients [134], [135]. However, the addition of cisplatin to re-irradiation and hyperthermia did not show clinical benefit compared with re-irradiation and hyperthermia alone in patients with inoperable locoregional recurrences [136].

### 5.2 Regional management of recurrent disease

#### 5.2.1 Clinically node-negative recurrent disease

In patients with cN0 ILRR, axillary lymph node dissection (ALND) is still performed [137], despite its limited role. In the 2021 St. Gallen Breast Cancer Consensus vote, only ∼12% of panelists supported completion ALND in this setting [138]. Given its associated morbidity [139], and comparable survival outcomes to repeat SLNB, its routine use is increasingly questioned [140]. Repeat SLNB is technically feasible [57], [140], [141], [142], [143], [144], although detection rates are lower than in the primary setting (71.9-81.0% after prior SLNB [145], [146], [147], [148], [149] and 38-55% after prior ALND [145], [147], [148], [149], [150]), largely due to altered lymphatic drainage patterns to less surgical accessible nodal sites (e.g. the IMC) [150]. Additionally, repeat SLNB results, whether positive or negative, do not appear to influence survival [57], [151], and even unsuccessful procedures are associated with low risk of subsequent regional or distant recurrence [145], [151], regardless whether an ALND is performed instead [145]. Collectively, these results support omission of (repeat) SLNB in selected cN0 ILRR, especially in patients with node-negative FDG PET/CT findings [57].

#### 5.2.2 Clinically node-positive disease recurrent disease

In clinically node-positive (cN+) ILRR, current guidelines recommend completion ALND after prior SLNB or resection of all detectable nodal disease after prior ALND [14], [127], [152]. However, NST may allow axillary re-staging and facilitate treatment de-escalation [14]. In primary BC, axillary re-staging techniques including SLNB, the MARI procedure (Marking an Axillary lymph node with a Radioactive Iodine seed) or a combination of both (targeted axillary dissection (TAD)) [153], have demonstrated feasibility and oncologic safety when guided by treatment response [154], [155], [156]. Data in ILRR remains limited, however, in a recent cohort study (n = 53) application of the MARI-procedure enabled omission of ALND in 87% of patients, with 81% only received axillary radiotherapy (ART) [157]. No axillary recurrences were observed, invasive disease free survival (iDFS) was 78% and OS was 83% after 44 months of follow up. These results support the potential for axillary re-staging and de-escalation in the recurrent setting.

For patients without axillary restaging, multimodality treatment remains the standard of care, combining surgery, radiotherapy and systemic therapy to improve survival outcomes [3], [14], [158], [159], [160], [161], [162], [163], [164], [165]. Surgical management typically involves completion ALND after prior SLNB, or removal of all detectable regional disease after prior ALND. However, less extensive surgical approach may be feasible. Konkin et al. reported no significant difference in 5-year disease-free survival (DFS) (36.6 vs. 41.3 months, p = 0.72) or OS (45.5 vs. 59.2 months, p = 0.26) between selective nodal removal and ALND for axillary recurrence [166]. Notably, combined surgery and radiotherapy significantly improved OS compared to either modality alone (p < 0.001). Accordingly, regional radiotherapy should be considered for all patients with regional recurrences [167], although re-irradiation poses similar challenges as in local disease.

In unresectable regional disease (e.g. supraclavicular or IMC nodal recurrences) treatment should consist of (re-)irradiation [14], [16], [17] and/or palliative systemic therapy tailored to prior systemic treatment and patient-specific factors [14].

### 5.3 Future perspectives

Although studies have identified patient subgroups eligible for repeat BCT, international guidelines provide limited direction on patient selection. In the absence of robust prospective evidence and evidence-based guideline recommendations, the recent international Delphi consensus study provides a valuable framework for selecting candidates. Despite increasing recognition and adoption of repeat BCT as an alternative treatment option, these recommendations have not yet been incorporated into major guidelines, and salvage mastectomy continues to be the standard approach.

As shown, re-irradiation results in acceptable toxicity rates and cosmetic results. However, a considerable proportion of patients still experiences adverse effects, and clinicians remain apprehensive to perform repeat BCT due to this risk. Multiple trials are currently evaluating postoperative PBI (Supplementary Table 2). In addition, two ongoing single-arm feasibility trials are investigating repeat BCT with pre-operative PBI in the recurrent setting (REPEAT NCT06640881, PAPBI-3 NCT06362616). When results are favorable, these trials will inform subsequent large, multicenter clinical trials assessing on long-term cosmetic, toxicity and oncological outcomes. The BREAST trial (NCT06954623) evaluates proton-based PBI as part of repeat BCT, while the RT-HYPE study (NCT06452485) assesses oncological outcomes and (patient-reported) treatment-related toxicity after re-irradiation with or without hyperthermia, including patient-reported toxicity.

Although omission of (repeat) SLNB in cN0 ILRR appears safe, particularly in low-risk, FDG PET/CT negative patients (cT1, grade 1-2, ER-positive and >50 years of age), the procedure remains commonly performed in clinical practice and recommended in guidelines. Three trials are currently evaluating repeat SLNB (Supplementary Table 2), while its role after NST remains unclear and requires further investigation.

In addition, patient selection and oncological safety of axillary re-staging techniques after NST have not been systematically evaluated in cN + ILRR. Axillary re-staging after NST may allow omission of ALND in selected cN + ILRR patients, as accurate assessment of nodal response may justify treatment with ART alone while preserving durable regional control.

To address these knowledge gaps, large prospective observational cohorts with harmonized data collection and long-term follow-up are needed. Such studies will enable robust evaluation of de-escalation strategies and inform future guideline development. For example the Korean SALT-LRR prospective registry study evaluates oncologic outcomes and toxicities of salvage treatment (Supplementary Table 2). Randomized trials (RCTs) in this setting may be increasingly difficult to justify, given that existing evidence already supports the safety of less invasive approaches as standard care comparator.

## 6 (NEO)ADJUVANT systemic treatment

Over the past decades, major advances in primary BC have improved survival through increasingly personalized systemic therapies, including the introduction of novel agents and the use of genomic assays (e.g. MammaPrint, Oncotype DX) to guide treatment de-escalation. In contrast, progress in the recurrent setting has been limited, with few prospective trials, no dedicated studies on NST, and limited evidence to inform treatment personalization. ILRR is associated with a substantial risk of DM, with a 5-year DDFS of 51-67% after local ILRR and 19-51.4% after locoregional recurrence [2], [165]. Prognosis is influenced by multiple factors, including subtype, grade, DFI and stage [168], [169]. Thus, effective systemic treatment to eradicate microscopic disease and improve survival is essential.

In the absence of solid data [168], [170], [171], [172], [173], expert opinion remains divided. Some reviews advocate tailoring treatment based on hormone receptor status [130], [171], [174], whereas others restrict systemic treatment to regional recurrences [173]. To support clinical decision-making, Lee et al. developed a risk stratification model to predict prognosis after ILRR, identifying cN + disease, DFI <30 months, and regional recurrence as independent predictors of survival outcomes [7]. Based on these factors, patients are classified into low-, intermediate-, and high-risk groups, providing a structured framework to guide systemic treatment decisions. However, despite its potential clinical utility, this model has not yet been widely adopted in routine practice. The optimal timing of systemic therapy also remains unclear. NST is recommended for unresectable recurrences to enable resection or radical radiotherapy [130], [168], [173], [174], but its broader role in ILRR is undefined [14], [16]. In current practice, treatment strategies are largely based on expert opinion, regimen extrapolation from early primary BC, prior systemic therapy, and DFI.

Evidence from RCTs is scarce. The CALOR trial [175] demonstrated a 10-year DFS benefit from chemotherapy in ER-negative ILRR (70% vs. 34% without), but not in ER-positive disease. However, several limitations hamper clear interpretation, including early termination (n = 162/997) likely related to randomization to a no-treatment arm, lack of molecular subtype stratification (HER2 testing was not mandatory), heterogeneous chemotherapy regimens, and regimens that were not contemporary with primary BC treatment. For example, only 18% of patients in the chemotherapy arm had received a contemporary prior chemotherapy regimen, including anthracyclines and taxanes. Despite these severe limitations, the CALOR trial is still referenced in guidelines in the absence of additional scientific evidence.

Similarly, the prematurely terminated SAKK23/82 trial (N = 167) [176] showed improved DFS of tamoxifen versus observation (5-year DFS rate of 61% vs. 33%), however, this difference was driven by fewer second local recurrences but not DM. In one-third of patients, ER status was unknown and patients with prior endocrine therapy were excluded. Consequently, both trials are outdated and severely underpowered with important methodological limitations, leaving several key questions unanswered. To date, no clinical trials have been conducted in HER2-positive ILRR.

The role of chemotherapy in ER-positive/HER2-negative ILRR remains unclear, as does the optimal regimen, particularly after prior treatment with third-generation chemotherapy. Similarly, uncertainty persists regarding the use of adjuvant endocrine therapy, including the optimal agent following prior treatment, treatment duration, and the potential role of Cyclin-dependent kinase 4/6 inhibitors (CDK4/6i) in this high-risk population. Evidence for immunotherapy in triple-negative ILRR and optimal systemic treatment strategies for HER2-positive disease are lacking.

Until new prospective data become available, (neo)adjuvant systemic therapy recommendations will continue to rely largely on expert opinion rather than robust scientific evidence.

### 6.1 Future perspectives

Research on systemic treatment for ILRR remains challenging and largely neglected. Given the general poor prognosis and substantial absolute number of patients affected annually, there is a clear unmet need for effective systemic therapies. Collaboration between academic and pharmaceutical partners is essential to facilitate dedicated ILRR trials to improve survival. However, slow patient accrual and premature termination of the CALOR and SAKK23/82 trials highlight the limited feasibility of trials including a non-treatment arm.

A key research priority is improving and implementing risk stratification models to identify patients with favorable prognosis who may safely omit or de-escalate systemic therapy. Models integrating clinicopathologic and molecular features, derived from large cohorts, may improve individualized decision-making. In this context, further validation of genomic assays, such as MammaPrint and Oncotype DX, in ILRR is warranted.

In addition, novel agents with efficacy in early-stage and metastatic BC should be evaluated in ILRR. For ER-positive disease, CDK4/6i have demonstrated benefit in high-risk early BC in the monarchE [177] and NATALEE [178] trials, and are currently investigated as an addition to standard endocrine therapy in ILRR (POLAR (NCT03820830); RAPhLRR (NCT05467891); AURA (NCT07190443) (Supplementary Table 2)). Oral selective estrogen receptor degraders (SERDs) (e.g imlunestrant [179]) may be particularly relevant in patients harboring activating estrogen receptor-1 (ESR1) mutations following prior aromatase inhibitor exposure [180]. Other emerging therapies, such as proteolysis targeting chimeras (PROTACs), potentially combined with CDK4/6i may further expand treatment options, particularly in endocrine therapy-resistant disease. For HER2-positive disease, antibody-drug conjugates (ADCs), such as trastuzumab-deruxtecan (T-DXd), have demonstrated substantial efficacy in previously treated HER2-positive metastatic BC [181], [182] and warrant evaluation in ILRR. In triple-negative disease, emerging strategies including anti-Trop2 ADCs [183], such as sacituzumab govitecan, and next-generation immune checkpoint inhibitors, represent promising therapeutic strategies.

In future trials, novel agents may be tested against a physician's-choice control arm consisting of standard, non-cross-resistant systemic therapy. The predictive value of pathological complete response (pCR) and circulating tumor DNA (ctDNA) clearance after NST in ILRR remains uncertain. If associated with survival outcomes, these markers may serve as surrogate endpoints to accelerate the evaluation of novel therapies in neoadjuvant trials.

## 7 Conclusion

ILRR remains a clinically challenging disease, with limited standardization of its definition, diagnostic work-up and optimal locoregional and systemic management. As summarized in this review, several key knowledge gaps remain (Table 3). Standardized criteria integrating molecular and clinical data should be established to reliably distinguish true ILRR from new primary tumors. Evidence-based recommendations for radiological staging, including the selective use of PET/CT according to ILRR subtype, are needed. Locoregional management requires further optimization, including clear eligibility criteria for repeat BCT and standardized re-irradiation strategies. In addition, the potential to omit repeat SLNB in cN0 ILRR and the role of axillary re-staging in cN + ILRR warrant further evaluation to support safe and effective treatment de-escalation. Finally, substantial efforts are needed to improve the poor prognosis of ILRR through more effective systemic therapy strategies. Collectively, these efforts are essential to establish evidence-based, multimodal guidelines and improve survival for patients with ILRR.

**Table 3:** Summary of knowledge gaps and future perspectives for isolated locoregional recurrent breast cancer.

	Knowledge gaps	Future studies/trials
**Definition**	a. Determine the optimal MCA technique b. Define clinical scenarios in which clonality testing is informative c. Establish clear thresholds and standardized molecular approaches for routine clinical use of MCA. d. Identify reproducible and valid clinicopathologic criteria to distinguish TR from SP BC. e. Determine the added value of statistical clonality models in clinical decision-making	a,b,c. Compare multiple MCA techniques (e.g. NGS, CNV, WES) in large unselected cohort of tumor pairs to identify the optimal method and implementation strategy in routine diagnostics d. Establish an unselected cohort of patients presenting with ipsilateral second breast cancer to compare clinical criteria against a molecular gold standard (preferably genomde-wide MCA or integrated statistical models). e. Evaluate the clinical utility of quantitative clonality models (e.g., Breakclone) in a large unselected cohort of tumor par
**Staging**	a. Identify which ILRR patients benefit from full body staging (by FDG-PET/CT). b. Define te role of FDG-PET/CT in regional staging c. Determine the optimal timing and indication for PET/CT (first-line vs. selective use) d. Evaluate the clinical utility of biologically targeted imaging (FAPI, FES and trastuzumab PET/CT)	a. Develop and validate risk prediction models for synchronous DM based on clinicopathologic characteristics b. Quantify the impact of FDG PET/CT on up- and downstaging and subsequent treatment modification in large ILRR cohorts c. Prospectively compare PET/CT with conventional imaging in risk-stratified populations d. Evaluate targeted PET tracers in subtype-specific ILRR cohorts
**Local treatment**	a. Prospectively identify eligibility criteria for repeat BCT b. Determine optimal re-irradiation strategies (technique, dose, fractionation and timing) c. Evaluate the long-term safety and efficacy of proton RT	a, b. Conduct large multicenter trials evaluating repeat BCT with standardized re-irradiation protocols (including PBI and preoperative RT) b. Compare re-irradiation techniques and timing in terms of oncologic outcomes, toxicity and cosmesis. b. Optimize dose and fractionation schedules through prospective trials
**Regional treatment**	a. Define the clinical value of repeat SLNB in cN0 ILRR, particularly after NST b. Evaluate the role and eligible patients of axillar re-staging techniques (MARI, TAD) in cN + ILRR	a.b. Establish large prospective real-world observational cohorts with harmonized data collection and long-term follow-up to: • Evaluate the omission of repeat SLNB (cN0); • Evaluate the oncologic safety of response-guided axillary re-staging strategies after NST in ILRR, and subsequent omission of ALND; • Compare tailored axillary approaches (ART vs ALND) based on response-guided axillary re-staging strategies after NST in ILRR
**Systemic therapy**	a. Identify optimal (neo)adjuvant systemic treatment options by subtype b. Determine the benefit of systemic therapy in risk-stratified ILRR populations c. Evaluate systemic treatment efficacy in patients with prior chemotherapy and/or endocrine therapy for the primary tumor (and possible treatment resistance) d. Establish the role of NST in resectable ILRR e. Identify surrogate endpoints associated with improved survival to serve as an early proxy for systemic treatment efficacy and biomarkers predictive of treatment response	a, b, c. Conduct trials comparing new agents to a physician's choice control arms with non-cross-resistant regimens by subtype. a, c. Evaluate novel agents in ILRR: • HER2-positive: HER2-targeted ADCs • Triple negative and luminal B: anti-Trop2 ADCs, next-generation ICI's • ER-positive: SERDs, PROTACs combined with CDK4/6i b. Validate genomic assays (e.g. MammaPrint, Oncotype DX) in ILRR cohorts e. Identify surrogate endpoints associated with survival, for example pCR and ctDNA clearance for neoadjuvant trials. d, e. Implement NST trials using surrogate endpoints as an early proxy for survival

Abbreviations: MCA, Molecular clonality analysis; TR, True recurrence; SP, Second primary; BC, Breast cancer; FDG-PET/CT, Fluorodeoxyglucose positron emission tomography/computed tomography; FAPI, Fibroblast activation protein inhibitor; FES, 16α-18Ffluoro-17β-oestradiol; BCT, Breast conserving therapy; RT, Radiotherapy; SLNB, Sentinel lymph node biopsy; cN0, Clinically node-negative; ILRR, Isolated locoregional recurrence; NST, Neoadjuvant systemic therapy; MARI, Marking an axillary node with a radioactive iodine seed; TAD, Targeted axillary dissection; cN+, Clinically node-positive; NGS, Next generation sequencing; CNV, Copy number variation; WES, ; PBI, Partial Breast Irradiation; ALND, Axillary lymph node dissection; ART, Axillary radiotherapy; ADC, Antibody-drug conjugate; ICI, Immune checkpoint inhibitor; ER, Estrogen receptor; SERD, Selective estrogen receptor degrader; PROTACs, proteolysis targeting chimeras; CDK4/6i, Cyclin-dependent kinase 4/6 inhibitor; pCR, Pathological complete response; ctDNA, circulating tumor DNA.

## CRediT authorship contribution statement

**Tess I.M. Snellen:** Writing – original draft, Methodology, Investigation, Conceptualization. **Charlotte M. Vervenne:** Methodology, Investigation, Writing – original draft, Conceptualization. **Vincent O. Dezentjé:** Writing – review & editing. **Terry Wiersma:** Writing – review & editing. **Astrid N. Scholten:** Writing – review & editing. **Esther H. Lips:** Writing – review & editing. **Marie-Jeanne T.F.D. Vrancken Peeters:** Writing – review & editing, Conceptualization, Supervision.

## Availability of data and materials

This article is a review of previously published studies and does not contain original research data.

## Ethics approval

Ethical approval was not required because this review did not involve human participants or the collection of patient data.

## Consent for publication

Not applicable. This review does not contain any individual person's data in any form.

## Funding

This research did not receive any specific grant from funding agencies in the public, commercial, or not-for-profit sectors.

## Declaration of competing interest

Given her role as Specialty Editor: Breast Surgery, Marie-Jeanne Vrancken Peeters had no involvement in the peer review of this article and had no access to information regarding its peer review. Full responsibility for the editorial process for this article was delegated to another journal editor.

Co-author V.O. Dezentjé serves on the advisory boards of AstraZeneca, Daiichi Sankyo, and Novartis.

All other authors declare they have no relevant financial or non-financial interest to disclose.

## Glossary

ACR: American College of Radiology
ADC: Antibody-drug conjugate
AI: Aromatase Inhibitor
ALND: Axillary lymph node dissection
APBI: Accelerated partial breast irradiation
ART: Axillary radiotherapy
BC: Breast cancer
BCS: Breast conserving surgery
BCT: Breast conserving treatment
CDK4/6i: Cyclin-dependent kinase 4/6 inhibitor
cN+: Clinically node-positive
cN0: Clinically node-negative
CNVseq: Copy number variation sequencing
CRT: Chemoradiotherapy
CT: Computer tomography
DFI: Disease free interval
ctDNA: circulating tumor DNA
DCIS: Ductal carcinoma in situ
DDFS: Distant disease free survival
DFS: Disease free survival
DM: Distant metastases
EBRT: External beam radiotherapy
ER: Estrogen receptor
ESR1: Estrogen receptor-1
FAPI: Fibroblast activation protein inhibitor
FDG: Fluorodeoxyglucose
FES: 16α-18Ffluoro-17β-oestradiol
FFPE: Formalin-fixed, paraffin-embedded
HER2: Human epidermal growth factor receptor 2
iDFS: Invasive disease free survival
ILRR: Isolated locoregional recurrence
IMC: Internal mammary chain
IORT: Intraoperative radiotherapy
LOH: Loss of heterozygosity
MARI: Marking an axillary node with a radioactive iodine seed
MCA: Molecular clonality analysis
MIBT: Multicatheter interstitial brachytherapy
MR(I): Magnetic resonance (imaging)
NCCN: National Comprehensive Cancer Network
NST: Neoadjuvant systemic therapy
NGS: Next generation sequencing
NTCP: Normal tissue complication probability
OS: Overall survival
PBI: Partial Breast Irradiation
pCR: Pathologic complete response
PET/CT: Positron emission tomography/computed tomography
PROTAC: Proteolysis targeting chimera
RCT: Randomized controlled trial
SERD: Selective estrogen receptor degrader
SLNB: Sentinel lymph node biopsy
SP: Second primary
TAD: Targeted axillary dissection
T-DXd: Trastuzumab-deruxtecan
TR: True recurrence
US: Ultrasound
WES: Whole exome sequencing
