# Supplementary material for: Knowledge gaps and future perspectives for the definition, diagnostics and treatment of isolated locoregional recurrent breast cancer
Source: Breast. 2026 Oct 21;90:104945. doi: 10.1016/j.breast.2026.104945 (PMC13743526; doi:10.1016/j.breast.2026.104945)
Supplement: Supplementary file 1 [file mmc1.docx]

Supplementary Table 1. Overview of eligibility criteria and risk factors for (accelerated) partial breast irradiation in primary breast cancer.

| Eligibility criteria | Correa et al., 2017  (ASTRO)* | Shaitelman et al., 2024  (ASTRO)* | Polgár et al., 2010  (GEC-ESTRO)* | Polgár et al., 2026  (GEC-ESTRO)* | Strnad et al., 2020  (DEGRO) |
| --- | --- | --- | --- | --- | --- |
| Eligible | • ≥50 years  • T1 disease (≤ 2 cm)  • Clear margins (≥ 2 mm)  • pN0  DCIS specific (low-risk)  • Screen detected  • Grade 1-2  • ≤ 2.5cm  • Clear margins≥ 3mm | • Grade 1-2 disease  • ER-positive  • Age ≥40 years  • Tumor size ≤2 cm | • ≥50 years  • Unifocal  • <3cm  • Non lobular  • No DCIS  • No EIC  • No LVI  • Negative margins (>2mm)  • pN0. | • >40 years • All invasive subtypes  • Associated LCIS allowed  • DCIS allowed  • Histological grade; any  • pTis-1-2 (≤30mm)  • Surgical margins: - Invasive: no tumor on ink  - DCIS: ≥2mm  • Unicentric  • Unifocal or multifocal (limited within 2cm)  • Focal LVI allowed  • HER2 status: any  • pN0 or pN1mi | • ≥50 years  • Invasive carcinoma (any grade) or DCIS (grade I-II)  • Tumor size ≤3cm  • Unifocal/unicentric  •Resection margins: - invasive: negative (≥2mm)  - invasive lobular or DCIS: negative (≥5mm)  • No LVI, no hemangiosis  • pN0/pNmi |
| Cautionary | • Age 40-49, if meeting all suitability criteria:  • Pathologic factors:  - Size 2.1-3.0 cm  - T2  - Close margins (<2 mm)  - Limited/focal LVI  - ER(-)  - Clinically unifocal with total size 2.1-3.0 cm  - Invasive lobular histology  - Pure DCIS ≤3 cm if criteria for "suitable" not fully met  - EIC ≤3 cm  - Close margins (<2cm)  DCIS specific  • ≤ 3 cm | • Grade 3 OR  • ER-negative  OR  • size >2 - ≤3 cm | • Clinical trials only | **-** | **-** |
| Contraindicated | • Age < 40 years  • Age 40-49 without meeting all suitability criteria  • Positive margins  DCIS specific  • >3 cm | • HER2-positive tumors not receiving anti-HER2 therapy  • LVI  • Lobular histology  Strong contradiction:  • cN+  • Positive margins  • Known BRCA1/2 mutations  • Age <40 years | • ≤ 40 years  • Multicentric disease  • > 3 cm  • LVI  • Extensive EIC  • Positive margins  • >4 nodes or Nx | • ≤ 40 years  • pT2 (>30 mm), pT3, pT4  • Surgical margins:  - Invasive: positive  - DCIS: <2mm  • Multicentric  • Multifocal (>2cm from the index lesion)  • EIC  • Extensive LVI  • TN subtype  • pNx ≥pN1a  • BRCA 1-2 gene mutation  • Neoadjuvant chemotherapy if used | • Stage IIB-IV breast cancer  • Non-assessable resection margins  • EIC  • Paget’s disease or skin involvement  • Age ≤ 40 years  • TN or HER2-positive subtype  • Neoadjuvant chemotherapy in treatment history |

* Both original and updated GEC‑ESTRO and ASTRO PBI classifications are presented. Original criteria are included due to their use in existing studies.
*Abbreviations: BRCA, breast cancer susceptibility gene; DCIS, ductal carcinoma in situ; ECI, extensive intraductal component; ER, estrogen receptor; HER2, human epidermal growth factor receptor 2; LCIS, lobular carcinoma in situ; LVI, lymphovascular invasion; TN, triple-negative.*
