# Supplementary material for: Knowledge gaps and future perspectives for the definition, diagnostics and treatment of isolated locoregional recurrent breast cancer
Source: Breast. 2026 Oct 21;90:104945. doi: 10.1016/j.breast.2026.104945 (PMC13743526; doi:10.1016/j.breast.2026.104945)
Supplement: Supplementary file 2 [file mmc2.docx]

Supplementary Table 2. Overview of (not yet) recruiting clinical trials in isolated locoregional recurrent breast cancer

| **Trial** | **NCT** | **About** |
| --- | --- | --- |
| **Local re-irradiation** | | |
| REPEAT | NCT06640881 | Preoperative Partial Breast Reirradiation and Repeat Breast-conserving Surgery in Patients With Recurrent Breast Cancer: the REPEAT Trial (REPEAT) |
| PAPBI-3 | NCT06362616 | Preoperative Accelerated Partial Breast Irradiation in Patients With Locally Recurrent or Second Primary Breast Cancer (PAPBI-3) |
| RT-HYPE | NCT06452485 | Postoperative Re-irradiaTion With and Without HYPERthermia: Toxicity, Quality of Life and Survival in Patients With Locoregional Recurrent Breast Cancer (RT-HYPE) |
|  | NCT06129747 | Repeat Breast Conserving Surgery Followed by Daily Partial Breast Irradiation in Ipsilateral Breast |
| RAY-no-BAN | NCT06704659 | Study on Recurrent Breast Cancer and Repeated Radiation Therapy (RAY-no-BAN) |
| PBReI | NCT05772390 | Partial Breast Re-irradiation in Women in Women with Locally Recurrent Breast Cancer Previously Treated with Conservative Surgery and Whole Breast Irradiation (PBReI) |
| PRESERVE | NCT05592938 | Partial Breast Re-irradiation Using Ultra Hypofractionation (PRESERVE) |
| BRASIL | NCT06867484 | Breast Re-irradiation After Second Ipsilateral Lumpectomy (BRASIL) |
| BREAST | NCT06954623 | Partial Breast Re-irradiation for Breast Cancer (BREAST) |
| **Repeat SLNB** | | |
|  | NCT04741737 | Repeat Sentinel Lymph Node Biopsy in Ipsilateral Breast Tumor Recurrence |
| RENATA | NCT06329089 | Reoperative Sentinel Lymph Node Biopsy in Patients With Recurrent Ipsilateral Breast Cancer (RENATA) |
|  | NCT06780748 | Photo-medicine-Guided Dual Approach for Reoperation of Sentinel Lymph Nodes in Locally Recurrent Breast Cancer Patients |
| **Systemic therapy** | | |
| POLAR | NCT03820830 | Evaluates the safety and efficacy of three years of palbociclib (CDK4/6i) in combination with standard endocrine therapy compared to standard endocrine therapy alone, focusing on iDFS in patients with ER-positive/HER2-negative resected ILRR |
| JCOG2313/AURA | NCT07190443 | Adjuvant Abemaciclib for Locoregional Recurrence of HR-positive, HER2-negative Breast Cancer (JCOG2313, AURA) |
| RaPhLRR | NCT05467891 | Ribociclib And Endocrine Treatment of Physician's Choice for Locoregional Recurrent, Resected Hormone Receptor Positive HER2 Negative Breast Cancer (RaPhLRR) |
| **Prospective registry** | | |
| SALT-LRR | NCT05933733 | Prospective registry to evaluate oncologic outcomes and toxicities of salvage treatment (surgery and/or RT) in patients with locoregional recurrent breast cancer. |
| **Not yet recruiting** | | |
| R_ReLoCC | NCT05406661 | Registry of Local Recurrences After Breast-conserving Surgery (R_ReLoCC) |
| - | NCT07668362 | Prospective Cohort Study of Local Recurrence After Radical Breast Cancer Resection |
| REPEAT | NCT07612254 | RE-irradiation With a Partial brEast, Accelerated Technique (REPEAT): A Single-Center, Multi-site, Prospective, Phase I/II Clinical Trial |

Trials were identified through a search of ClinicalTrials.gov (<https://clinicaltrials.gov>).
